# Aerobic, resistance and combined training for adults with chronic kidney disease: A protocol for a systematic review and network meta-analysis

**DOI:** 10.1097/MD.0000000000023518

**Published:** 2020-12-04

**Authors:** Rong Zeng, Honghao Lai, Zhuoyan Li, Beibei Chen, Lu Wang, Yali Zhang

**Affiliations:** a Department of Nephrology, the Second Hospital; b School of Public Health, Lanzhou University; c The First Clinical Medical College of Lanzhou University, Lanzhou, China.

**Keywords:** aerobic exercise, chronic kidney disease, combined exercise, exercise, resistance exercise

## Abstract

**Background:**

Chronic kidney disease (CKD) as a disease that poses a great threat to human health, which has become a public health issue of great concern. Studies have found that exercise training has a positive effect on improving the condition of chronic kidney disease. We will conduct a network meta-analysis to assess the effects of aerobic training, resistance training and combined aerobic and resistance training in treating CKD patients.

**Methods:**

We will search PubMed, EMBASE, Medline, Cochrane Central Register of Controlled Trials (CENTRAL), and Web of science to identify randomized control trails (RCTs) that assessed the effect of different exercise training for CKD patients. Cochrane Handbook will be used to evaluate the risk of bias of included articles. We will use Stata or R software to perform data analysis.

**Results and Conclusion:**

Our systematic review and network meta-analysis will be the first study that investigates the effect of different exercise training for CKD patients, and will provide evidence for management of chronic kidney disease.

**Ethics and dissemination:**

The data involved in this study are from published articles. For this reason, there is no need for ethical approval or patient consent.

**Trial registration:**

the registration number was: CRD42020157280

## 1 Introduction

Chronic kidney disease (CKD) is a disease that causes irreversible damage to the function and structure of the kidney in several months or years for various reasons. Common causes include diabetes, hypertension and glomerulonephritis. High incidence in high-income and middle-income countries, the incidence can reach 10%. According to the definition and classification for chronic kidney disease proposed by the National Kidney Foundation Kidney Disease Outcomes Quality Initiative (NKF-KDOQI) in 2002, we can know that Renal injury or low glomerular filtration rate (GFR o60 ml/min per 1.73 m^2^) for more than three months, chronic kidney disease can be diagnosed without considering the cause. According to the different levels of GFR, it can be divided into 5 types.^[1–3]^ Chronic kidney disease is often characterized by high mortality and increasing risk of cardiovascular disease. With the development of chronic kidney disease, the quality of life of patients has declined dramatically, and even their lives have been threatened. The personal and family consequences of chronic kidney disease are catastrophic. At the same time, it has also caused a serious burden on society. Therefore, chronic kidney disease became a major public health problem to be solved urgently.^[4,5]^

For most patients with chronic kidney disease, drug therapy, dialysis and surgery are the main methods. But some studies have found that exercise can be used as a means of treating chronic kidney disease. In these studies, patients with chronic kidney disease were divided into three approximate cohorts: pre-dialysis, dialysis and transplant, and exercise intervention was carried out. It was found that exercise played a positive role in them.^[6–8]^ This is because exercise has a targeted positive effect on a range of risks of chronic kidney disease, such as chronic inflammation, cardiovascular disease and diabetes. Exercise also has beneficial effects on many risk factors for chronic kidney disease, such as hypertension. The greatest effect of exercise on patients with chronic kidney disease is to improve their cardiovascular health and vascular function.^[9–11]^ According to relevant research, exercise can effectively reduce diastolic and systolic blood pressure of pre-dialysis patients and dialysis patients.^[12,13]^ Three months of home-based exercise also had a significant effect on reducing arterial stiffness.^[10]^ Exercise can improve the arterial compliance of patients and reduce the risk of cardiovascular disease.^[14]^ Continuous regular exercise promotes an anti-inflammatory environment and may improve disease status.^[15]^ It is certain that any form of exercise is beneficial to patients with chronic kidney disease, but the best way to exercise for patients with chronic kidney disease has not been determined.^[16]^ We focus on the two most acclaimed exercise interventions, aerobic and resistance exercises, and combined training of the two exercises. In some studies, it has been found that aerobic exercise has no effect on the corresponding outcome indicators, so it is difficult to explain the role of aerobic exercise in patients with chronic kidney disease.^[17]^ In one study, after 35 weeks of aerobic training, both glomerular filtration rate and exercise endurance were positively affected in patients with chronic kidney disease.^[18]^ Studies have shown that chronic kidney disease patients after resistance exercise training, muscle strength significantly increased, diastolic blood pressure also decreased.^[19]^ Few studies have been conducted on these issues, and the conclusions are controversial. There is a lack of network meta-analysis of these three currently. So we try to compare the curative effect and difference between them with network meta-analysis. Through targeted network meta-analysis, combined with traditional direct and indirect comparisons, we can find the most effective and safe interventions for different patients. It is of great significance for different patients to choose appropriate treatment measures.

Our study aims to evaluate the effects of aerobic exercise, resistance exercise and combined exercise training on chronic kidney disease patients in different situations by meta-network analysis.

## 2 Methods and analysis

### 2.1 Registration

This systematic review and network meta-analysis has been registered on the International Prospective Register of Systematic Reviews (PROSPERO). The registration number is: CRD42020157280.

### 2.2 Search strategy

We will conduct systematical searches of PubMed, EMBASE, Medline, Cochrane Central Register of Controlled Trials (CENTRAL), and Web of science. We will not limit the year and language of publication. We will also track the references of relevant systematic review and meta-analyses for additional studies. The keywords for searching articles are as followings: Chronic kidney disease, exercise, aerobic exercise, resistance exercise, combined exercise, physical activity.

### 2.3 Inclusion criteria

#### 2.3.1 Type of participants

Patients aged over 18 years old with primary chronic kidney disease that meet diagnostic criteria will be considered. Studies on animals, children, adolescents or pregnant women will be excluded.

#### 2.3.2 Type of design

We will include randomized controlled trials (RCTs) comparing aerobic, resistance and combined training for patients with chronic kidney disease. In order to avoid possible sources of heterogeneity, we will exclude cluster-randomized trials and cross-over trials.

#### 2.3.3 Type of interventions

We will only consider 4 exercise training modalities: aerobic exercise training, resistance exercise training, combined aerobic training and resistance training (combined exercise) and no exercise. Studies involving any other interventions will not be considered.

#### 2.3.4 Type of outcomes

We will consider blood lipids, blood pressure, heart rate, muscle morphology and morphometrics, and health-related quality of life as the primary outcomes; and the Physical fitness, Compliance, and adverse event will be considered as the secondary outcomes.

### 2.4 Definition of interventions

Aerobic exercise training: We defined aerobic exercise as a regimen containing aerobic components performed at least three to five times per week for at least four weeks and performing minimum for 30mins each time. Aerobic components included walking, cycling, jogging, and swimming but not limit to these types.^[20]^

Resistance exercise training: We defined resistance exercise as exercise performed against some type of progressive resistance on a minimum of two days each week to increase their muscle strength, muscle endurance or muscle power.^[21]^ Resistance components included bench press, seated row, shoulder press, leg press, and weight strength but not limit to these types.

Combined exercise: Participant performed the aerobic training program plus the resistance training program to assure an adequate dose of each type of exercise.

No exercise: Participant were asked do not to participant in any type of exercise and were asked to revert to their level of activity at baseline and maintain their current lifestyle or the RCTs do not mention whether people in control group do some exercise.

### 2.5 Study selection

We will use Endnote X8 software to manage the literature search records. A pilot-literature selection will be conducted to ensure high inter-rater reliability among the reviewers. According to eligibility criteria teams of two reviewers will screen title and abstract of all the retrieved bibliographic records independently. Any differences that arise during the screening process will be discussed or resolved by a third reviewer. Articles that have been screened will be stored and kept uniformly.

### 2.6 Data extraction

A standard data collection forms will be created for reviewers to use when extract data. After pilot testing of data extraction, teams of paired reviewers will independently extract the fowling data: basic information of the article, including the first author, the name of the published journal, the year of publication, the country of publication, the type of publication; population characteristics, including gender, average age, age range, source (outpatient, hospitalized, community), inclusion criteria, baseline status of other stratified factors and number of withdrawals/dropouts; intervention characteristics, including the type of intervention, the specific measures of intervention, the frequency of intervention, the duration of each intervention; and outcomes of interest.

### 2.7 Risk of bias of individual studies

The assessment of risk of bias in individual study will be assessed using the Cochrane Handbook version 5.1.0.,^[22]^ which including the method of adequate sequence generation, allocation concealment, blinding of participants and personnel, incomplete outcome data, selective reporting, and other sources of bias (e.g., extreme baseline imbalance and funding source). Two reviewers will assess the risk of bias of each study independently. Any differences arising from the evaluation process will be discussed and handed over to a third reviewer. We will use three indicators of low risk, high risk and unclear bias risk to classify methodological quality.

### 2.8 Data analysis

For dichotomous variable, we will calculate the results as ratio ratios (RRs) for each study and its 95% confidence interval (CI). For continuous variable, mean difference (MD) with the relative 95%CI will be calculated when the study used the same instruments for assessing the outcome. Standardized mean difference (SMD) will be used when studies used different instruments. *P* value < .05 will be considered as statistically significant.

We will use the function of ‘networkplot’ function of STATA 15.1 to generate network plots to describe and present the geometry of different form of exercise training. And a Frequentist network meta-analysis will be performed using the ‘netmeta’ of R software to assess the homogeneity in the whole network, the homogeneity within designs, and the homogeneity/consistency between designs. The inconsistency between direct and indirect comparisons will be assessed using node-splitting method.^[23]^ We will also calculate the treatment ranking according to *P* scores, which were based solely on the point estimates and standard errors of the network estimates.

## Author contributions

**Conceptualization:** Rong Zeng.

**Investigation:** Rong Zeng, Beibei Chen.

**Methodology:** Rong Zeng, Hong hao Lai, Beibei Chen, Lu Wang.

**Project administration:** Rong Zeng, Beibei Chen.

**Resources:** Zhuo Yan Li, Lu Wang, Ya Li Zhang.

**Supervision:** Rong Zeng, Hong hao Lai, Zhuo Yan Li.

**Validation:** Hong hao Lai, Ya Li Zhang.

**Visualization:** Hong hao Lai.

**Writing – original draft:** Rong Zeng, Hong hao Lai.

**Writing – review & editing:** Rong Zeng, Beibei Chen.
